# Environmental Conditions and Mite Vectors Shape the Spatiotemporal Patterns of Scrub Typhus in Guangdong Province, Mainland China

**DOI:** 10.3390/tropicalmed10110326

**Published:** 2025-11-20

**Authors:** Peiwei Fan, Tian Ma, Ze Meng, Fangyu Ding, Shuai Chen, Mengmeng Hao, Jiaqi Li, Jun Zhuo, Jiping Dong, Wenqi Xie, Qian Wang, Tingting Kang, Kai Sun, Genan Wu, Yongqing Bai, Canjun Zheng, Dong Jiang

**Affiliations:** 1 Institute of Geographic Sciences and Natural Resources Research, Chinese Academy of Sciences, Beijing 100101, China; fanpeiwei0244@igsnrr.ac.cn (P.F.); mat.19b@igsnrr.ac.cn (T.M.); chenshuai@igsnrr.ac.cn (S.C.); haomm@igsnrr.ac.cn (M.H.); lijiaqi241@mails.ucas.ac.cn (J.L.); zhuojun6545@igsnrr.ac.cn (J.Z.); dongjiping2017@igsnrr.ac.cn (J.D.); xiewenqi2523@igsnrr.ac.cn (W.X.); jiangd@igsnrr.ac.cn (D.J.); 2 College of Resources and Environment, University of Chinese Academy of Sciences, Beijing 100049, China; 3 School of Engineering, Xizang University, Lhasa 850001, China; mengze@igsnrr.ac.cn; 4 Nuffield Department of Medicine, Centre for Tropical Medicine and Global Health, University of Oxford, Oxford OX3 7LF, UK; qian.wang@stx.ox.ac.uk; 5 Mahidol Oxford Tropical Medicine Research Unit, Faculty of Tropical Medicine, Mahidol University, Bangkok 10400, Thailand; 6 School of Urban Planning and Design, Shenzhen Graduate School, Peking University, Shenzhen 518055, China; kangtt@pku.edu.cn; 7 GeoAI Lab, Department of Geography, University at Buffalo, Buffalo, NY 14214, USA; ksun4@buffalo.edu; 8 Institute of Spacecraft Application System Engineering, China Academy of Space Technology, Beijing 100094, China; genanw425@126.com; 9 State Key Laboratory of Remote Sensing Science, Aerospace Information Research Institute, Chinese Academy of Sciences, Beijing 100094, China; baiyq@aircas.ac.cn; 10 Chinese Center for Disease Control and Prevention, Beijing 102206, China; zhengcj@chinacdc.cn

**Keywords:** scrub typhus, spatiotemporal patterns, driving factors, bimodal pattern, Bayesian hierarchical mixed model

## Abstract

Scrub typhus has emerged as a life-threatening and increasingly prevalent vector-borne disease. While the spatial and temporal distributions of scrub typhus have been studied by the research community, the main driving factors that influence the spatiotemporal patterns of the disease remain under investigation. Using Guangdong Province as a case study, we combined monthly scrub typhus case data from 2010 to 2019 and environmental and vector-related datasets with a Bayesian hierarchical mixed model to elucidate the spatiotemporal characteristics of the disease. This study revealed that the most highly endemic areas of scrub typhus are concentrated in the western and southern parts of Guangdong Province. A distinct bimodal pattern of scrub typhus was observed, with peaks typically occurring from May to July and October to November. The fitted model indicated that forest, cropland, and chigger mites were positively associated with scrub typhus transmission. Furthermore, climate and vectors were identified as key factors shaping the bimodal seasonal patterns of scrub typhus. Despite data-related limitations, including the treatment of population as a time-invariant variable and the binary simplification of vector suitability, sensitivity analyses confirmed the robustness of the model, offering valuable insights for scrub typhus prevention in Guangdong.

## 1. Introduction

Scrub typhus is a climate-sensitive vector-borne infectious disease transmitted through the bites of chigger mites [1,2]. The disease is typically characterized by sudden onset of high fever and the formation of eschar at the site of the mite bite, which, in severe cases, may progress to multiple organ failure and even death [3,4]. Although scrub typhus was historically confined to the Asia–Pacific region, recent reports have documented its emergence in other parts of the world, including Africa [4], South America [5], Europe [6], and the Middle East [7]. Unfortunately, there is currently a lack of rapid diagnostic tools and effective vaccines [8]. With over one billion individuals at risk and an estimated one million cases of scrub typhus occurring annually, scrub typhus is gradually posing a certain threat to public health globally [9,10].

China is one of the traditional endemic regions of scrub typhus, with documented evidence of the disease dating back thousands of years [11]. Before the 1980s, scrub typhus was endemic mainly in south of the Yangtze River in China [12]. Since then, the endemic range has progressively expanded northward, with increasing numbers of cases reported in provinces such as Shandong, Shanxi, and Hebei [13]. In 2006, scrub typhus was reinstated as a notifiable disease in China’s national surveillance system, and its incidence has risen sharply in recent years [14]. By 2022, surveillance data indicated that more than 180,000 cases had been reported nationwide.

Guangdong Province, as the first region to isolate and report scrub typhus cases in China, is one of the most severely affected endemic areas, exhibiting distinct seasonal and spatial incidence clustering patterns [15,16]. These patterns are considered to be related to climate factors such as temperature, precipitation, and relative humidity [17,18]. For example, Wei et al. used time series Poisson regression models to establish associations between climate factors and scrub typhus in Guangzhou and reported that temperature and precipitation were positively correlated with scrub typhus [17]. Similarly, Huang et al. adopted correlation analysis and a random forest model to discover a lagged positive relationship between scrub typhus incidence rates and precipitation in Guangzhou [18]. Moreover, environmental factors such as elevation and land use are believed to influence the occurrence of scrub typhus by affecting the habitat of chigger mites and human activities, such as fieldwork and outdoor activities [19,20,21,22]. As the exclusive vector of scrub typhus, the chigger mite plays an essential role in sustaining and amplifying disease transmission [23]. Previous studies have indicated that the growth and development cycles and species abundance of vectors can influence the occurrence and spread of diseases [24,25].

Given the high burden of scrub typhus in Guangdong, it is essential to identify the key drivers underlying its spatiotemporal dynamics. However, comprehensive studies that quantify the seasonal and spatial patterns of scrub typhus in relation to environmental factors and vector suitability are still lacking. To address this gap, we aim to quantify the seasonal and spatial patterns of scrub typhus across 123 counties of Guangdong from 2010 to 2019 and assess associations with lagged climate, land cover, and vector suitability index using Bayesian spatiotemporal model. Our findings demonstrate that the environment and vectors are key factors that shape the spatiotemporal patterns of scrub typhus in Guangdong, which is important for effectively targeting timely treatment programs and vector control activities in areas with high rates of scrub typhus seasonal transmission.

## 2. Materials and Methods

### 2.1. Data

#### 2.1.1. Scrub Typhus Cases

The surveillance dataset on human cases of scrub typhus in China, spanning from 2010 to 2019, was obtained from the Chinese Center for Disease Control and Prevention (China CDC, https://www.phsciencedata.cn/Share/, accessed on 10 January 2025). To ensure patient confidentiality, all individual-level data were deidentified before analysis. The data only included the report address of patients, dates of illness onset, and case classifications (confirmed, clinically diagnosed, suspected), without including any personal patient information. The dataset was processed under professional guidance to exclude suspected cases and remove duplicates. The final dataset included all clinically diagnosed and laboratory-confirmed cases, incorporating cases with delayed reports or those with corrected information based on the date of illness onset. In total, 43,272 clinically diagnosed or laboratory-confirmed scrub typhus cases reported in Guangdong Province from 2010 to 2019 were included in this study.

#### 2.1.2. Climate Data

Climate factors play key roles in the transmission of scrub typhus, which influences the occurrence and spread of the disease through various pathways and, to some extent, determines the prevalence and seasonality of the disease [26,27,28,29,30]. For example, Ding et al. demonstrated that climatic factors such as temperature, precipitation, and relative humidity were the key driving factors for the spatiotemporal patterns of scrub typhus in southern China [29]. Han et al. suggested that average temperature and relative humidity have a significant effect on scrub typhus, exhibiting a pattern of initially increasing and then decreasing effects [30]. Thus, we selected temperature (°C), precipitation (mm), and relative humidity (%) as important covariates in this study. The monthly mean climate records of weather stations in China for temperature (°C), precipitation (mm), and relative humidity (%) from 2010 to 2019 were collected from the average daily data of the China Meteorological Data Service Center (http://data.cma.cn, accessed on 10 January 2025), from which 1 km × 1 km gridded data were generated via ANUSPLIN-SPLINA version 4.36 software.

#### 2.1.3. Land Cover and Altitude Data

Several studies have shown that habitat complexity and the diversity of land cover have significant impacts on scrub typhus [22,31,32,33,34]. For example, Liu et al. revealed that scrub typhus is more likely to occur in croplands, forests, and grasslands than in other land use types [22]. To analyze the associations between key land use types and scrub typhus risk, we reclassified the Moderate Resolution Imaging Spectroradiometer (MODIS, MCD12Q1.v6, 2010–2019; NASA, Washington, DC, USA) land cover data into six major categories (Table S1), consistent with the Chinese land resource management framework. Additionally, a machine-readable reclassification rule file is available as part of the Supplementary Material to facilitate replication. We subsequently selected the coverages of forests, grasslands, and croplands as covariates because of their significance in the habitat distribution of chigger mites and vector-human interaction levels, and their proportions accounted for more than 70% on average in Guangdong [22]. In addition, altitude is considered an important factor that affects the distribution of scrub typhus [32]. For example, Zheng et al. [32] reported that altitude is an important driving factor of scrub typhus in southern China. Therefore, we adopted altitude as a covariate. The gridded terrain data were downloaded from the Data Center for Resources and Environmental Sciences, Chinese Academy of Science (RESDC) (http://www.resdc.cn, accessed on 10 January 2025), with a spatial resolution of 1 km × 1 km.

#### 2.1.4. Habitat Suitability Index of Chigger Mites

The transmission risk of scrub typhus is directly influenced by the habitat suitability index of chigger mites, which serve as the primary vectors responsible for disease transmission [35,36,37,38,39]. In China, *Leptotrombidium deliense* (*L. deliense*) and *Leptotrombidium scutellare* (*L. scutellare*) are the two main dominant species transmitting scrub typhus [38,39]. The habitat suitability maps for *L. deliense* and *L. scutellare* were obtained from a previous study [35], which employed an ensemble boosted regression tree (BRT) modeling approach. Specifically, the models were developed at the county level across mainland China using georeferenced occurrence records of both mite species [36] integrated with contemporary environmental covariates. More details on the sources of data are provided elsewhere [35,36]. The environmental suitability layers were subsequently divided into high (labelled as 1) and low (labelled as 0) layers using a threshold value of 0.5 (Figure S1), following the definition adopted in the original suitability dataset [35].

#### 2.1.5. Population

We obtained population density data from 2015 from the Resources and Environmental Science Data Registration and Publication System (http://www.resdc.cn, accessed on 10 January 2025), with a spatial resolution of 1 km × 1 km.

### 2.2. Spatiotemporal Modeling Approach

To avoid the impact of COVID-19 (travel restrictions and medical problems since 2020) on reported cases of scrub typhus, we focused our study from 2010 to 2019. We employed a spatiotemporal Bayesian hierarchical mixed model on the basis of monthly cases of scrub typhus during this period to explore the environmental and vector-related factors driving scrub typhus occurrence in Guangdong, mainland China. To address the issue of numerous zero cases observed in the counties of Guangdong, the occurrence of scrub typhus cases ($y_{st}$) was assumed to follow a zero-inflated negative binomial (ZINegBin) distribution (Equation (1)).

(1) $$y_{st}\sim ZiNegbin\left(\mu_{st},\;k\right)$$

(2) $$log\left(\mu_{st}\right)=\log\left(P_{st}\right)+\sum \beta_{i}x_{i,st}{{+{\;m}_{t}+Y+u}_{s}+v}_{s}+\alpha$$

We modelled the mean ($\mu_{st}$) number of monthly (*t* = 1 to 120) scrub typhus cases for 10 years (2010–2019) in each county (*s* = 1 to 123) of Guangdong via Equation (2). The log population ($P_{st}$) was included as an offset, and the cases ($\log\left(\mu_{s,t}\right)$; Equation (2)) were estimated by a combination of spatiotemporal covariates. Covariates were selected based on the climate sensitive of scrub typhus and the central role of chigger mites in its transmission [29,40]. $\sum \beta_{i}x_{i,st}$ for covariates included climate variables (temperature, precipitation, relative humidity), land cover (forest, grassland, cropland), the habitat suitability index for chigger mites (*L. deliense* and *L. scutellare*), and altitude. We assessed multicollinearity among all covariates using the variance inflation factor (VIF), with all VIF values below 5 (Table S2), indicating no substantial multicollinearity among predictors. $\beta_{i}$ is the coefficient corresponding to each covariable. The term α denotes the intercept. To capture potential nonlinear relationships between climatic factors and scrub typhus incidence, we modelled temperature, precipitation, and relative humidity using second-order random walk (RW2) smoothers in INLA. Climatic, altitude, and land cover variables were standardized, while habitat suitability indices of chigger mites were expressed on a 0–1 scale to ensure comparability of effects. Default log-gamma priors (shape = 1, rate = 0.00005) were used for the precision (smoothing) hyperparameters.

In addition, we included spatiotemporal random effects in our model framework to account for unobserved confounding factors and to capture unknown variability in the data. Unexplained variation might occur due to unmeasured factors, such as vector control and population movements. Random effects for the month were specified in our model ($m_{t}$) by introducing a first-order random walk latent model to capture any seasonality in scrub typhus cases in Guangdong [41]. To construct reasonable models, unstructured random effects with the same distribution were specified for each year (Y, 2010–2019) in assumption of independence for each year [41]. To allow for spatial correlation in scrub typhus cases across counties in Guangdong, we assigned conditional intrinsic Gaussian autoregressive model priors to the spatial random effects ($u_{s}$). The adjacency structure was derived from county-level shapefiles using queen contiguity, and the spatial random effects were modelled using the Besag-York-Mollié 2 (BYM2) specification with default scaling and penalized-complexity (PC) priors for both the precision and mixing parameters [42]. We also specified independent diffuse Gaussian exchange priors ($v_{s}$) for each county to account for any additional uncorrelated variation in scrub typhus cases across counties in Guangdong that could not be measured [42].

All covariates in our models were scaled by subtracting the covariate mean from each value and dividing by the covariate standard deviation. Model fit was assessed via Bayesian methods of model comparison, the deviance information criterion (DIC), and the Watanabe-Akaike information criterion (WAIC) to gauge the effectiveness and robustness of our modelling approach [43]. Lower DIC and WAIC values indicate better model effects. All analyses were conducted in R (version 4.3.1). Model coefficients were exponentiated to obtain relative risks, and the 95% Bayesian credible intervals (CrIs) were used to express uncertainty. Bayesian hierarchical models were implemented using the Integrated Nested Laplace Approximation (INLA) approach (INLA version 22.12.18; https://www.r-inla.org, accessed on 10 January 2025).

## 3. Results

### 3.1. Spatiotemporal Pattern of Scrub Typhus

**Figure 1:**
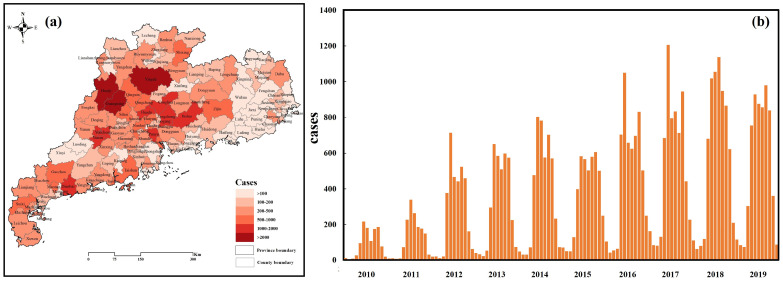
Spatiotemporal distribution of reported scrub typhus cases in Guangdong Province from 2010 to 2019. (**a**) The geographical distribution of cases at the county level. (**b**) The monthly numbers of scrub typhus cases from 2010 to 2019 in Guangdong Province. All map boundaries corresponded to the official administrative boundaries of Guangdong Province (2010), derived from the Guangdong Platform for Common GeoSpatial Information Services.

From 2010 to 2019, a total of 43,272 scrub typhus cases were reported in Guangdong, China. The yearly count of new cases in Guangdong has been steadily increasing, from 1111 in 2010 to 6263 in 2019 (Figure S2a). The geographical pattern of scrub typhus at the county level is mapped in Figure 1a, which shows that the cases exhibited spatial clustering, with the western and northern regions of Guangdong being high-incidence areas. The top 10 counties in Guangdong Province with the highest incidence rates of scrub typhus are detailed in Table S3, revealing a notable upward trend in the cumulative number of cases over the study period (Figure S2b). The highest number of cases, 3211 (7.42% of total cases), was reported in Huaiji, followed by 2795 cases in Guangning (6.46% of total cases), and 2313 cases in Yingde (5.34% of total cases). Moreover, we observed a distinct bimodal seasonal pattern, with the first peak occurring generally from May to July and the second peak occurring from October to November (Figure 1b).

### 3.2. Distribution of Scrub Typhus Cases Under Different Environmental and Vector Conditions

**Figure 2:**
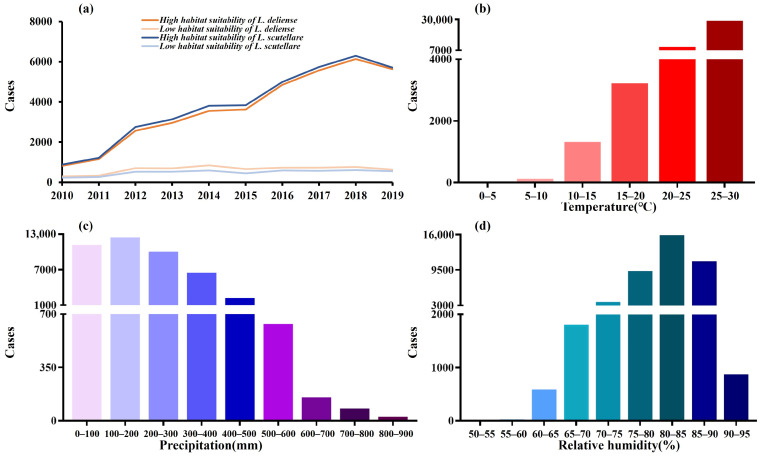
The distribution of scrub typhus cases varies across different environmental and vector conditions. (**a**) Habitat suitability of chigger mites (colors indicate suitability levels). (**b**) Temperature. (**c**) Precipitation. (**d**) Relative humidity.

The distribution of scrub typhus cases under different environmental and vector conditions is shown in Figure 2. There were more scrub typhus cases in areas with high habitat suitability for chigger mites, including both *L. deliense* and *L. scutellare*, than in areas with low habitat suitability in Guangdong (Figure 2a). In terms of the relationship between temperature and the number of cases (Figure 2b), the cases were concentrated in the temperature range of 10–30 °C, and the number of cases increased with increasing temperature. According to the relationship between precipitation and the number of cases (Figure 2c), the vast majority of cases occurred when the precipitation was between 0 and 600 mm. For relative humidity (Figure 2d), the cases were concentrated in the range of relative humidity exceeding 60%, and the number of cases was increased with increasing. The distribution of scrub typhus cases across land use types and altitudes is shown in Figure S3.

### 3.3. The Spatiotemporal Modelling of Scrub Typhus

**Figure 3:**
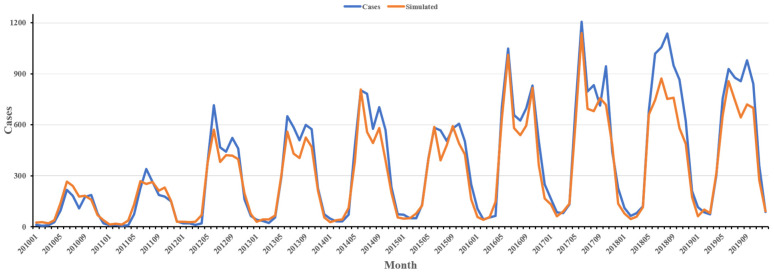
Monthly number of scrub typhus cases in Guangdong (2010–2019). Observed data (blue) and simulations from the best-fitting model (orange).

In this study, we constructed a ‘Base model’ that solely incorporates random effects (Table 1). We subsequently expanded this base model to include nine influencing factors, including forest (F), grassland (G), and cropland (C), temperature (TEM), precipitation (PRE), and relative humidity (RHU), and the habitat suitability of chigger mites *L. deliense* (Ld) and *L. scutellare* (Ls), resulting in the full model (Table 1). Specifically, for the climate factors (temperature, precipitation, and relative humidity), we considered lag times ranging from 0 to 4 months, yielding a total of 125 combinations. Model performance was compared using DIC and WAIC. Our findings indicate that the model achieved the highest accuracy when precipitation was lagged by three months, whereas temperature and relative humidity did not lag, with a DIC of 55,350.95 and a WAIC of 55,360.94. Details of the top-ranked models are provided in the Supplementary Materials (Table S4). The results of the optimal model simulation are highly consistent with the actual values, and show a consistent bimodal trend with the original cases as depicted in Figure 3. As a sensitivity check, we replaced the static 2015 population offset with annual county-level populations from 2010–2019, and conducted additional analyses using a continuous suitability index (Table S4). These sensitivity analyses yielded similar conclusions, indicating that the main results were robust.

**Figure 4:**
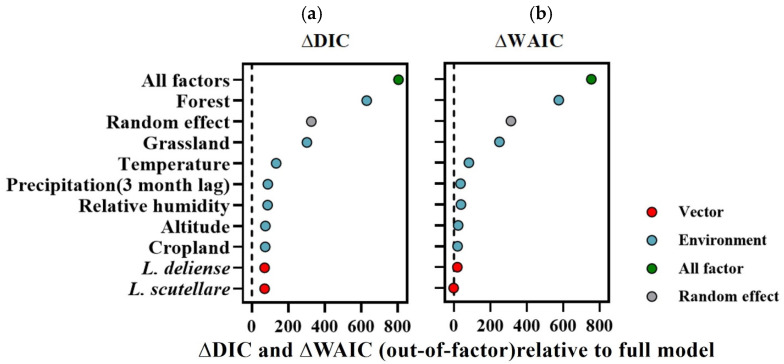
The impact of environmental and vector factors on the spatiotemporal prediction of scrub typhus. ∆DIC and ∆WAIC were used to compare the accuracy of different models, thereby representing the impact of each variable on the model through the difference in model accuracy. (**a**) ∆ DIC. (**b**) ∆WAIC. On the *Y*-axis, each candidate model excludes one covariate at a time from the best-fitted model. The points depict changes in DIC and WAIC relative to the best-fitted model (dashed lines). The colour of the points signifies the out-of-covariate category: environmental factor (blue), vector factor (red), all factors (green), and random effect (grey). A value higher than zero indicates that excluding the factor increases the spatiotemporal variability of the model (i.e., decreases the ability to explain the unexplained portion) compared with the best-fitted model, whereas a value lower than zero indicates the opposite.

To estimate the individual predictive impact of each covariate, we systematically excluded one covariate at a time from the best-fitted model (Figure 4). We compared the accuracy of different models using ∆DIC and ∆WAIC to represent the impact of each variable on the model. The results indicate that all the factors are crucial for the model, as their removal led to increases of 803.54 and 753.56 in DIC and WAIC, respectively. Specifically, compared with the best-fitting model, the exclusion of random effect factors reduced its ability to explain unexplained variance. Among the environmental factors, the covariate with the greatest influence was forest, followed by grassland and temperature. Subsequently, precipitation (3-month lag), relative humidity, altitude, and farmland also play significant roles. While the inclusion of vector factors exerts a lower influence on model fitting than do other environmental or random effects, its incorporation also serves to enhance model precision to a certain degree.

### 3.4. The Driving Factors on the Spatiotemporal Dynamics of Scrub Typhus

**Figure 5:**
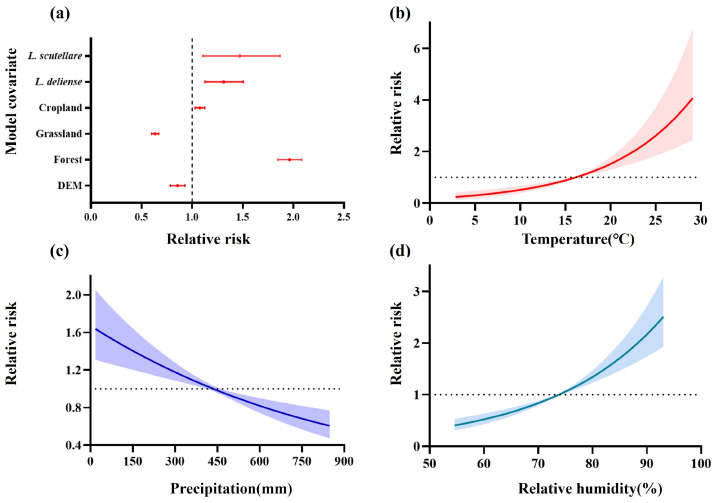
Environmental and vector factor drivers of scrub typhus in Guangdong Province. Relative risk (points) and 95% credible intervals (CIs, horizontal lines) for the associations between scrub typhus incidence and (**a**) land cover, altitude and vector factors. The relative risk (solid lines) and 95% CIs (shading) for the nonlinear effects of temperature (**b**), precipitation (**c**), and relative humidity (**d**), respectively. All reported values (exp (β)) represent partial effects estimated from the Bayesian hierarchical mixed model, controlling for other covariates.

We identified a positive association between the number of scrub typhus cases and the habitat suitability of *L. deliense* and *L. scutellare*; specifically, the relative risk of *L. deliense* on scrub typhus was 1.05 (95% CrI [0.89–1.25]), and the relative risk of *L. scutellare* on scrub typhus was 1.36 (95% CrI [1.05–1.73]) (Figure 5a). Our results indicated that land cover types were associated with scrub typhus. A positive association was observed between scrub typhus and forest and cropland, whereas grassland exhibited a negative relationship. Specifically, the forest relative risk was 2.02 (95% CrI [1.90–2.14]), the cropland relative risk was 1.06 (95% CrI [1.01–1.11]), and the grassland relative risk was 0.66 (95% CrI [0.62–0.69]) (Figure 5a). In our final model, the effects of temperature, precipitation, and relative humidity were modeled using non-linear smooth terms (RW2) to capture potential non-linear relationships. The effect of climate on scrub typhus in Guangdong Province was assessed by specifying climate variables, including temperature, precipitation, and relative humidity (Figure 5b–d). The results showed that temperatures between approximately 16 °C and 30 °C were associated with an increased transmission risk of scrub typhus, while temperatures below 16 °C were linked to a lower risk. Precipitation demonstrated a positive association with the incidence of scrub typhus up to about 450 mm, beyond which the association became negative. Similarly, when relative humidity exceeded 75%, the risk of scrub typhus increased, whereas lower humidity was associated with reduced incidence. Quantitative analysis showed that when temperature increased from the 25th to the 75th percentile, the relative risk of scrub typhus incidence was 5.25 (95% CrI: 2.94–10.45), while the corresponding relative risks for precipitation and relative humidity were 1.09 (95% CrI: 0.81–1.46) and 0.89 (95% CrI: 0.69–1.17), respectively (Table S5).

## 4. Discussion

We mapped the spatiotemporal distribution of scrub typhus in Guangdong Province from 2010 to 2019 and observed significant spatial clustering and a distinct bimodal seasonal pattern. By constructing a Bayesian hierarchical spatiotemporal model, we integrated climatic, land cover, elevation, and vector suitability variables to quantify the drivers of the disease’s spatiotemporal heterogeneity. Climatic variables showed nonlinear effects, with precipitation exhibiting a lag effect of approximately three months. Forest cover was positively associated with the incidence of scrub typhus (2.02 of relative risk, 95% CrI [1.90–2.14]), grassland was negatively associated (0.66, 95% CrI [0.62–0.69]), and cropland showed a moderate positive association (1.06, 95% CrI [1.01–1.11]). The habitat suitability of *L. deliense* and *L. scutellare* was also linked to an increased risk of scrub typhus (1.05, 95% CrI [0.89–1.25]; and 1.36, 95% CrI [1.05–1.73], respectively)

The main clusters were concentrated in the western and northern regions of Guangdong, with Huaiji, Guangning, and Yingde counties showing the highest number of cases. These counties also have large proportions of forest and cropland areas, consistent with our finding that increased suitability of forest and cropland environments was associated with higher infection risk. This result aligns with previous studies [21,44,45], suggesting that forested and agricultural environments provide favorable habitats for chigger mites and that farmers are more likely to be exposed to mite bites during fieldwork. Model comparison indicated that including a 3-month lag for precipitation improved model fit, suggesting a delayed suppressive effect possibly related to the chigger life cycle. For instance, the average life cycle of *L. deliense* is about 89 days [46], with all stages except larvae living within the topsoil [47]. Moderate rainfall may maintain suitable soil moisture over several months, facilitating mite development [35,48]. In contrast, excessive rainfall immediately scours the topsoil and destroys the habitat of eggs and other life stages [49], leading to a reduction in the larval population that emerges approximately three months later, thereby reducing the risk of human infection.

The simulated results reproduced the observed bimodal seasonal pattern (Figure 3), with the first peak in summer (around June) and the second in autumn (around September). We found strong evidence that this pattern is consistent with the combined influence of climate and vector dynamics. The nonlinear increase in disease risk above approximately 16 °C and relative humidity above 75% is consistent with findings from entomological [32,50] and modeling studies [17]. Previous research has shown that *L. deliense* and *L. scutellare* differ in environmental suitability [14,35,46,51]. *L. deliense* develops and reproduces optimally at 18–28 °C and humidity above 80% [46], whereas *L. scutellare* prefers relatively cooler and drier conditions [35]. When climatic conditions are unsuitable, mite activity declines [46]. Consequently, *L. deliense* tends to be active and reproduce in summer, when temperature and humidity are favorable, contributing to the first seasonal peak [51]. Meanwhile, *L. scutellare* begins to reproduce during the cooler autumn and winter months, accounting for the second peak [14]. In addition, human activity patterns may further reinforce this bimodal distribution [40]. Surveillance data indicate that farmers, who engage in intensive agricultural work during spring and autumn, are the most affected occupational group (Figure S4).

Several limitations should be acknowledged. First, our model did not account for time-varying population dynamics and used a threshold approach for vector suitability, which may introduce potential bias. Although we conducted multiple sensitivity analyses demonstrating the robustness and interpretability of our model, some degree of uncertainty remains. Second, as there is currently no available vaccine for scrub typhus, timely detection and treatment are particularly crucial for elderly populations. However, assessing how treatment and reinfection influence the spatiotemporal transmission dynamics remains challenging.

Despite these limitations, our findings highlight the combined influence of climate, land cover, and vector suitability in shaping the spatial and temporal patterns of scrub typhus in Guangdong, offering valuable insights for targeted prevention and vector control strategies.

## 5. Conclusions

This study analyzed the spatial and temporal distribution of scrub typhus in Guangdong Province and investigated how environmental and vector-related factors influence disease transmission by using a Bayesian hierarchical mixed model. From 2010 to 2019, we observed distinct spatial clustering patterns and bimodal seasonality of scrub typhus across the province. The model results demonstrated that climate conditions and vector mites were important factors associated with both the spatial clustering and bimodal seasonality of scrub typhus. Furthermore, different land cover types significantly influenced transmission risk, with forests and croplands facilitating disease spread. These findings highlighted the importance of considering environmental conditions and mite vectors when formulating comprehensive intervention plans against scrub typhus outbreaks, which could assist public health authorities in devising targeted prevention and control strategies.

## Figures and Tables

**Table 1:** Formulae of spatiotemporal Bayesian model.

Model	Description
Base model	$log\left(\mu_{st}\right)=\log\left(P_{st}\right){{+m_{t}+Y+u}_{s}+v}_{s}+\alpha$
Full model (m = 0, 1, 2, 3, 4)	$log\left(\mu_{st}\right)=\log\left(P_{st}\right)+\beta_{TEM}{TEM}_{s,t-m}+\beta_{RHU}{RHU}_{s,t-m}+\beta_{PRE}{PRE}_{s,t-m}+\beta_{F}F_{st}+\beta_{G}G_{st}\;\;\;\;\;\;\;+\beta_{C}C_{st}+\beta_{Ld}{Ld}_{st}+\beta_{Ls}{Ls}_{st}{{+m_{t}+Y+u}_{s}+v}_{s}+\alpha$

## Data Availability

The original contributions presented in this study are included in the article/Supplementary Material. Further inquiries can be directed to the corresponding author.

## Supplementary Materials

The following supporting information can be downloaded at: https://www.mdpi.com/article/10.3390/tropicalmed10110326/s1, Figure S1: Habitat suitability of *L. deliense* and *L. scutellare* by county in Guangdong Province. Figure S2: Cumulative number of scrub typhus cases each year from 2010 to 2019. Figure S3: The distribution intervals of various variables and the number of scrub typhus cases. Figure S4: The time trend of cases was divided by (a) age and (b) occupation in 2010–2019. Table S1: IGBP land cover reclassification. Table S2. Variance inflation factor (VIF) values for covariates included in the model. Table S3: The top ten counties with scrub typhus cases from 2010 to 2019 in Guangdong. Table S4: The performance of the spatiotemporal Bayesian model. Table S5. Standardized effects of climatic variables on scrub typhus incidence.
