# Supplementary material for: Environmental Conditions and Mite Vectors Shape the Spatiotemporal Patterns of Scrub Typhus in Guangdong Province, Mainland China
Source: Trop Med Infect Dis. 2025 Nov 20;10(11):326. doi: 10.3390/tropicalmed10110326 (PMC12656344; doi:10.3390/tropicalmed10110326)
Supplement: Supplementary file 1 [file tropicalmed-10-00326-s001.zip › tropicalmed-3909443-supplementary.pdf]

Additional file:

Environmental conditions and mite-vector shape the spatiotemporal patterns of scrub typhus in Guangdong province, mainland China

Additional Figures

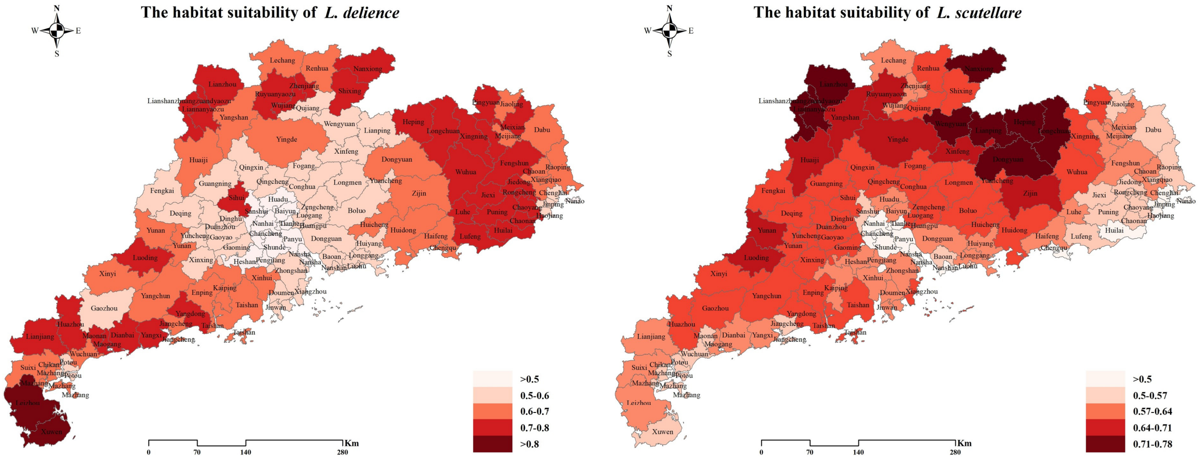

Figure S1. Habitat suitability of *L. deliense* and *L. scutellare* by county in Guangdong Province. Generally, a value less than 0.5 is considered low risk, while a value greater than 0.5 denotes high risk.

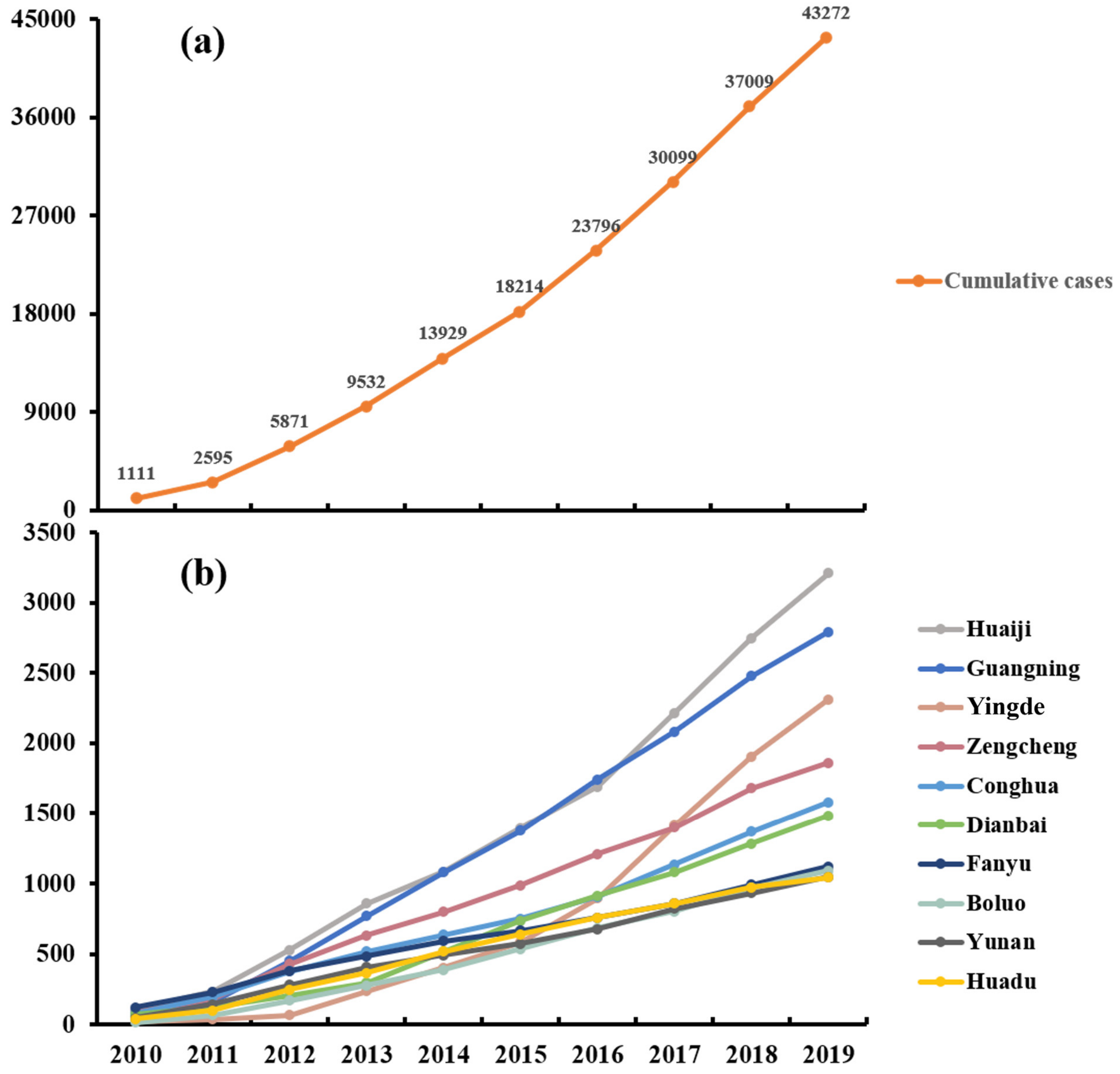

Figure S2. Cumulative number of scrub typhus cases each year from 2010 to 2019. (a) Guangdong province and (b) the top 10 counties for cases.

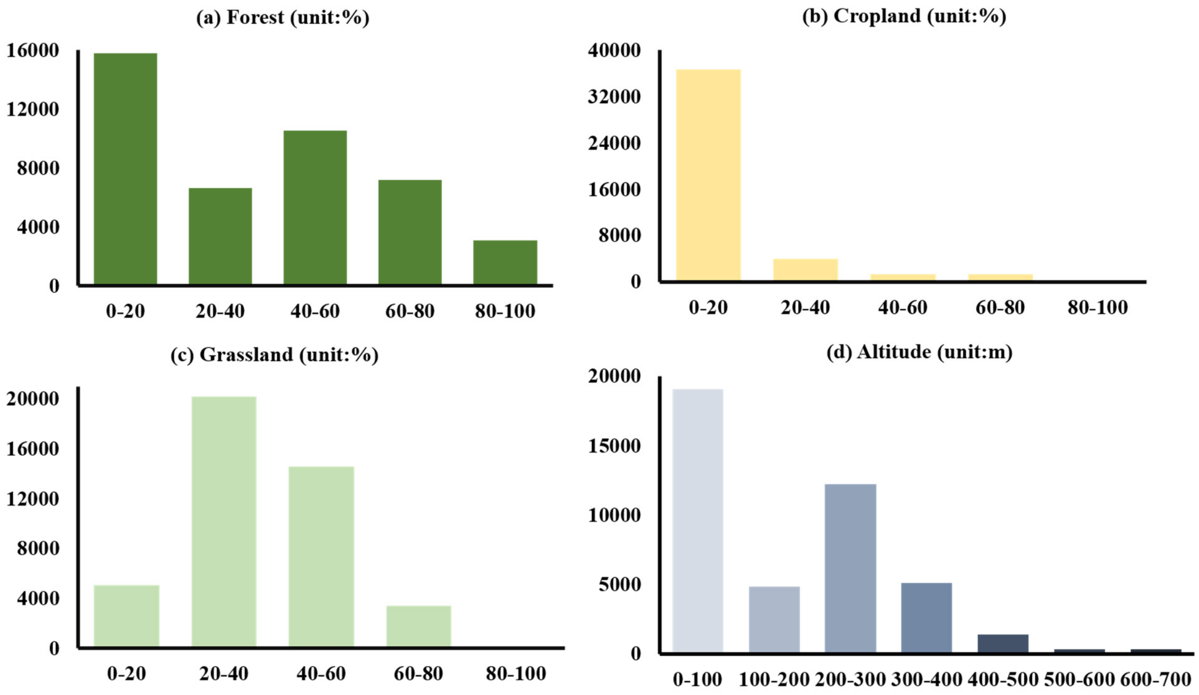

Figure S3. The distribution intervals of various variables and the number of scrub typhus cases. (a) Forest, (b) Cropland, (c) Grassland, and (d) Altitude.

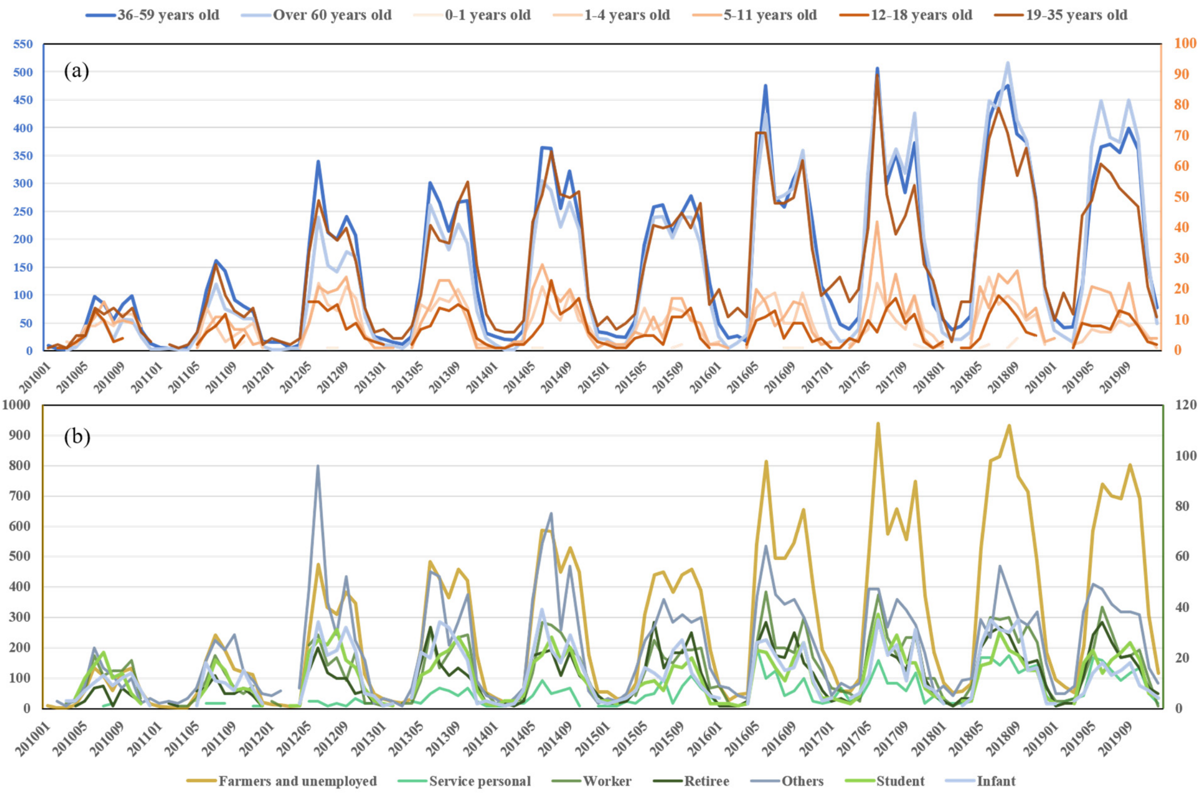

Figure S4. The time trend of cases was divided by (a) age and (b) occupation in 2010-2019.

Additional Tables

Table S1. IGBP land cover reclassification. The MODIS (Moderate Resolution Imaging Spectroradiometer) land cover data (MCD12Q1.v6) product provides an annual distribution of land cover types with a resolution of 500 meters. The MCD12Q1.v6 contains 17 major land cover types according to the International Geosphere-Biosphere Programme (IGBP). The land use types utilized in the study were identified through a process of reclassification.

IGBP	Description	Reclassification
Evergreen Needleleaf Forests	Dominated by evergreen conifer trees (canopy >2m). Tree cover > 60%.	Forest
Evergreen Broadleaf Forests	Dominated by evergreen broadleaf and palmate trees (canopy > 2m). Tree cover > 60%.	
Deciduous Needleleaf Forests	Dominated by deciduous needleleaf (larch) trees (canopy>2m). Tree cover > 60%.	
Deciduous Broadleaf Forests	Dominated by deciduous broadleaf trees (canopy >2m). Tree cover > 60%.	
Mixed Forests	Dominated by neither deciduous nor evergreen (40-60% of each) tree type (canopy > 2m). Tree cover >60%.	
Closed Shrublands	Dominated by woody perennials (1-2mheight) > 60% cover.	
Open Shrublands	Dominated by woody perennials (1-2mheight) 10-60% cover.	
Woody Savannas	Tree cover 30-60% (canopy > 2m).	
Savannas	Tree cover 10-30% (canopy > 2m).	Grassland
Grasslands	Dominated by herbaceous annuals (<2m)	
Cropland/Natural Vegetation Mosaics	Mosaics of small-scale cultivation 40-60% with natural tree, shrub, or herbaceous vegetation.	Cropland
Croplands	At least 60% of area is cultivated cropland.	
Urban and Built-up Lands	At least 30% impervious surface area including building materials, asphalt, and vehicles.	Urban
Barren	At least 60% of area is non-vegetated barren (sand, rock, soil) areas with less than 10% vegetation.	Barren
Water Bodies	At least 60% of area is covered by permanent water bodies.	Water
Permanent Snow and Ice	At least 60% of area is covered by snow and ice for at least10 months of the year.	
Permanent Wetlands	Permanently inundated lands with 30-60% water cover and >10% vegetated cover.	

Table S2. Variance inflation factor (VIF) values for covariates included in the model.

Covariates	VIF value
**Precipitation**	2.82
**Temperature**	2.34
**Relative humidity**	2.63
**DEM**	1.73
**Forest**	2.68
**Grassland**	1.46
**Cropland**	1.37
L. deliense	2.78
L. scutellare	1.37

Note: All VIF values were below 5, indicating no substantial multicollinearity among predictors.

Table S3. The top ten counties with scrub typhus cases from 2010 to 2019 in Guangdong. The cumulative number of cases in these top 10 counties accounted for more than 40%, while the remaining 113 counties accounted for less than 60%.

County	Year										Total	Proportion (%)
	2010	2011	2012	2013	2014	2015	2016	2017	2018	2019		
Huaiji	107	125	297	329	227	308	299	523	534	462	3211	7.42
Guangning	55	104	292	319	311	294	369	339	395	317	2795	6.46
Yingde	20	17	29	168	170	180	311	518	493	407	2313	5.35
Zengcheng	85	100	244	203	168	187	221	191	281	180	1860	4.30
Conghua	79	116	182	139	121	114	155	230	234	210	1580	3.65
Dianbai	72	52	80	92	221	218	177	167	205	201	1485	3.43
Panyu	121	109	149	103	111	74	91	99	134	131	1122	2.59
Boluo	13	51	104	107	113	148	146	120	165	124	1091	2.52
Yunan	48	97	136	124	88	79	104	143	114	112	1045	2.41
Huadu	41	59	146	121	149	125	115	101	116	70	1043	2.41

Table S4. The performance of the spatiotemporal Bayesian model. The full model listed in the top ten with the lowest DIC.

**Model**	**The month lag**	**Offset**	**Vectors (*L. deliense* and *L. scutellare*)**	**DIC**	**WAIC**
Base model	--	2015 population	Binary	56092.28	56103.84
Full model	RHU:0, TEM:0, PRE:3	2015 population	Binary	55350.95	55360.94
	RHU:2, TEM:2, PRE:3	2015 population	Binary	55354.86	55368.39
	RHU:2, TEM:0, PRE:3	2015 population	Binary	55355.22	55365.43
	RHU:0, TEM:2, PRE:3	2015 population	Binary	55355.49	55368.53
	RHU:1, TEM:2, PRE:3	2015 population	Binary	55356.29	55370.21
	RHU:1, TEM:0, PRE:0	2015 population	Binary	55358.07	55368.82
	RHU:3, TEM:0, PRE:3	2015 population	Binary	55361.28	55370.02
	RHU:0, TEM:0, PRE:4	2015 population	Binary	55361.53	55371.19
	RHU:0, TEM:0, PRE:0	2015 population	Binary	55363.74	55373.72
	RHU:1, TEM:0, PRE:4	2015 population	Binary	55366.85	55377.76
Sensitivity model	RHU:0, TEM:0, PRE:3	Annual population (2010–2019)	Binary	55463.65	55469.93
	RHU:0, TEM:0, PRE:3	2015 population	Continuous values	55492.68	55502.06

Table S5. Standardized effects of climatic variables on scrub typhus incidence. The interquartile (25th–75th percentile) relative risks (RRs) were estimated from the Bayesian spatiotemporal model.

Covariates	Lag (months)	25th percentile	75th percentile	Incidence Rate Ratio	Lower 95% Crl	Upper 95% Crl
**Precipitation**	0	-0.85	0.93	5.25	2.94	10.45
**Temperature**	3	-0.83	0.6	1.09	0.81	1.46
**Relative humidity**	0	-0.57	0.71	0.89	0.69	1.17
